# Recent Progress in the Development of Diagnostic Tests for Malaria

**DOI:** 10.3390/diagnostics7030054

**Published:** 2017-09-19

**Authors:** Francis D. Krampa, Yaw Aniweh, Gordon A. Awandare, Prosper Kanyong

**Affiliations:** 1 West African Centre for Cell Biology of Infectious Pathogens (WACCBIP), University of Ghana, Legon, Accra, Ghana; fkrampa@gmail.com (F.D.K.); aniweh@gmail.com (Y.A.); gawandare@ug.edu.gh (G.A.A.); 2 Department of Biochemistry, Cell & Molecular Biology, University of Ghana, Legon, Accra, Ghana; 3 Nanotechnology & Integrated Bioengineering Centre, Ulster University, Jordanstown BT37 0QB, UK

**Keywords:** rapid diagnostic tests (RDT), biosensing, lateral flow assays, *Plasmodium* spp., multiplex biomarker detection, histidine-rich protein 2 (HRP2), lactate dehydrogenase (LDH), aldolase, point-of-care tests (POCT), disposal medical devices, infectious diseases

## Abstract

The impact of malaria on global health has continually prompted the need to develop effective diagnostic strategies. In malaria endemic regions, routine diagnosis is hampered by technical and infrastructural challenges to laboratories. These laboratories lack standard facilities, expertise or diagnostic supplies; thus, therapy is administered based on clinical or self-diagnosis. There is the need for accurate diagnosis of malaria due to the continuous increase in the cost of medication, and the emergence and spread of drug resistant strains. However, the widely utilized Giemsa-stained microscopy and immunochromatographic tests for malaria are liable to several drawbacks, including inadequate sensitivity and false-positive outcomes. Alternative methods that offer improvements in performance are either expensive, have longer turnaround time or require a level of expertise that makes them unsuitable for point-of-care (POC) applications. These gaps necessitate exploration of more efficient detection techniques with the potential of POC applications, especially in resource-limited settings. This minireview discusses some of the recent trends and new approaches that are seeking to improve the clinical diagnosis of malaria.

## 1. Introduction

The launch of several initiatives to eradicate malaria [1] has resulted in a global decline in morbidity and mortality [2]. Yet malaria remains a major global health problem, especially in tropical regions, and in 2015 an estimated 218 million cases with 395,000 deaths were recorded in Africa [3]. Among non-endemic regions such as European countries and the US, cases of imported malaria are on the increase [4]. One of the factors that has ensured the persistence of malaria has been the lack of analytical sensing tools that allow for early and accurate detection in asymptomatic individuals with low parasitemia levels in peripheral blood [5]. At present, the World Health Organization (WHO) recommends case management to be guided by detecting *Plasmodium* parasites or antigens in the peripheral blood of febrile patients and asymptomatic carriers. However, current techniques including microscopy and rapid diagnostic tests (RDTs) do not have satisfactory sensitivity for parasitemia. Alternative methods with superior performance are relatively expensive with low throughput; thus, rendering them unsuitable for routine use. Therefore, there is the need for the development of effective diagnostic strategies for field application, where diagnostic expertise in malaria is often lacking [6,7]. New technologies are focusing on developing point-of-care (POC) tests that afford improvement in all test parameters; these could help strengthen laboratory diagnostics in resource-limited malaria endemic areas.

This minireview highlights some of the efforts seeking to improve or develop new diagnostic techniques for the detection and diagnosis of malaria. These include high throughput immunochemical assays, nucleic acid detection techniques, biomarker identification and biosensing approaches with or without microfluidic channels; these techniques allow for the identification of disease-specific biomarkers rather than the simple documentation of the presence of the pathogen.

## 2. Current Clinical Diagnostic Methods

The diagnostic tools currently available for the identification of *Plasmodium* spp. include light and fluorescence microscopy, RDTs such as immunochromatographic lateral flow assays [8], serology, Quantitative Buffy-Coat (QBC) concentration, and nucleic acid amplification techniques such as Polymerase Chain Reaction (PCR) and isothermal amplification; which are extensively reviewed elsewhere [9]. The algorithm for laboratory diagnosis involves identifying the causative *Plasmodium* spp. in Giemsa-stained microscopy or its antigens with an RDT.

## 3. Advances in the Identification of Diagnostic Biomarkers for Malaria

New insights into disease specific markers have also been the hallmark of clinical malaria diagnostics. Diagnostic biomarkers can be genomic, transcriptomic, proteomic or metabolomic markers [10]. In malaria, qualitative and quantitative analysis of biomarkers may facilitate the determination of parasite species, estimation of parasitemia, intensity of the immune response and prognostic information. Most of the focus on biomarkers in malaria diagnosis has been blood and/or serum-based. Recent advances in proteomics have also focused on the proteome from other body fluids such as saliva and urine to identify potential disease-related markers [11,12]. Specific biomarkers detectable at the early stages of malaria infection and their detection methods are summarized in Table 1.

## 4. Advances in the Detection of Malaria Biomarkers in Clinical Samples

### 4.1. Non-blood Based Assays

Almost all malaria diagnostic methods rely on blood or its products for the detection of the disease. In cases where repeated sampling is required, the invasive processes involved in specimen collection are frequently associated with poor compliance [28]. Where specimen from less invasive procedures show high diagnostic accuracy, *Plasmodium* infections can be more accurately investigated with minimal inconvenience to patients. Trace amounts of malaria parasite DNA have been detected in urine and saliva from some malaria-infected individuals, however, it is unclear whether the amount of parasite DNA present in these body fluids is representative of the parasite load in peripheral blood [29].

According to Nwakanma et al., saliva is a promising non-invasive approach for detecting malaria infection [30]. They reported sensitivity and specificity of 73% and 97% respectively, when nested PCR (nPCR) amplification of the multicopy 18S rRNA gene in saliva was compared with blood-film microscopy. The sensitivity further increased to 82% at high parasitemia (≥1000 parasites/µL). Quantitative PCR (qPCR) results for saliva also correlated significantly with microscopy counts. An earlier study that used nPCR to target the mitochondrial cytochrome *b* gene (*cytb*) of parasites in matched blood, saliva and urine from malaria patients demonstrated a significant improvement in the diagnostic performance of *cytb* in saliva and urine over blood-film microscopy [31].

A recently developed Urine Malaria Test™ (UMT) dipstick that detects *P. falciparum* Histidine-Rich Protein 2 (*Pf*HRP2) showed moderate level of sensitivity when compared with blood-film microscopy in a normal field setting with a sensitivity of 83.75% and specificity of 83.48% [32]. The UMT showed an improved sensitivity compared to other studies [33] with a detection limit of 120 parasites/μL; this 50% increase in sensitivity was partly attributed to the *Pf*HRP2 concentration in the urine at the time of sampling [30,34,35]. The authors recommended optimizing the amount of antibody impregnated into the device as well as the quantity of the specimen required. Previous reports have also suggested the use of first void morning urine as it might contain higher antigen titers than samples taken at later times [33].

### 4.2. Blood-Based Assays

#### Nucleic Acid Detection Techniques

New molecular approaches to parasite detection seek to reduce cost, Therapeutic Turnaround Time (TTAT) and labor intensity, where the conventional nested 18S rRNA PCR assay has fallen short in clinical application. Despite being highly sensitive and specific, the nPCR assay is laborious, relatively time consuming and provides only qualitative data. These drawbacks make it unsuitable for routine first-line screening or field application especially in resource-limited settings. An ideal molecular assay must be low-cost and have the capacity to screen large number of samples with ease and within a short TTAT. Quantitative molecular methods such as real-time PCR have relatively short assay preparation and analysis time and as such, better suited for large-scale studies than nPCR assays. Moreover, they are relatively less expensive due to the use of small volumes of “low-cost” primer/fluorophore [36]. A summary of various molecular approaches employed for the clinical diagnosis of malaria are provided in Table 2.

More recently, a transcription-based amplification system was developed for quantitative nucleic acid sequence-based amplification (QT-NASBA) [39] that targets specific RNA. Other techniques such as Loop-mediated isothermal amplification (LAMP) [52] and photo-induced electron transfer (PET)-PCR [46] have the potential to revolutionize the patient’s experience, as results can be obtained within minutes; leading to shorter waiting hours during hospital visits. Lucchi et al. [37] also demonstrated that multiplexed photo-induced electron transfer (PET)-PCR, which relies on self-quenching primers for the detection of *Plasmodium* spp. is robust and much more cost-effective than nPCR [36,37,46]. The assay has a detection limit of 3.2 parasites/µL; which is deemed to be satisfactory for testing a large number of samples, as it only cost USD 2.0 per test (versus USD 3.2 for nPCR) [37].

LAMP is the most widely researched isothermal diagnostic technology that has been used to identify all the human *Plasmodium* spp. [39,53]. A commercially available Loopamp Kit (Eiken Chemical Co. Ltd, Tokyo, Japan) detects *P. falciparum* [54] and enables the indirect detection of *P. vivax* using a combination of pan-genus and *P. falciparum* specific LAMP primers [55]. Optimization of the LAMP platforms described by Goto et al. [56] has been adopted for the identification of *P. falciparum* as a high-throughput technique [57]. Its limit of detection for the *Plasmodium* genus is 2.5 parasites/μL from whole blood and 25.0 parasites/μL from dried blood spots and is relatively less expensive per test. This offers a cost-effective molecular diagnostic platform in resource-limited settings. A combination of LAMP with a lateral flow device has been developed to target dihydrofolate reductase thymidylate synthase (*dhfr-ts*) genes of *P. falciparum* and *P. vivax* in blood or extracted DNA [58]. It consists of a streptavidin-biotin reaction between hybridized LAMP amplicons and gold-labelled anti-FITC antibodies on the lateral flow device strip that allows visualization of the result [58]. However, this rapid molecular diagnostic device has not been validated on clinical samples and is limited in sensitivity at low parasitemia level. Another LAMP-based assay, the Non-Instrumented Nucleic Acid (NINA)-LAMP has been used for the detection of *Plasmodium* spp. in blood samples from malaria-suspected patients [40]. It uses an exothermic chemical reaction between saline and a magnesium iron alloy to generate energy for amplification within an insulated thermos flask-like device. Unlike the LAMP and lateral flow device combination, this assay was validated in Ethiopia utilizing primers for amplification of parasite mitochondrial DNA (Loopamp™ malaria Pan/Pf detection kits (Eiken Chemicals Co. Ltd, Tokyo, Japan)) [40]. The NINA-LAMP showed high sensitivity of 96.8% in the diagnosis of malaria as well as 100% differentiation of non-*falciparum* species. These performance characteristics have a diagnostic accuracy superior to microscopy and are comparable to nPCR [40]. The self-generating energy, lack of post-processing handling and fast TTAT make it attractive for POC diagnostic applications. A LAMP assay for *P. vivax* α-tubulin was also developed using six primers that recognize the targeted gene at different regions [47]. The sensitivity of this assay in field samples with suspected malaria was found to be 100% (95% CI, 96.4–100%). When microscopy and RDT was used to analyze the same field samples, sensitivity values of 75.0% (95% CI, 66.8–81.7%) and 93.0% (95% CI, 87.9–96.4%), were found for microscopy and RDT, respectively. This further demonstrates its viability for POC biomedical applications.

## 5. Application of Bio-Sensing Technology in Malaria Diagnosis

Biosensors are self-contained analytical devices which can analyze complex matrices such as blood and urine without the need for additional processing steps or reagent addition, in contrast to other bioanalytical systems [59].

A cell based label-free electrochemical biosensor that detects parasitized red blood cells has been developed by Kumar et al. [51]. In this study, a monoclonal antibody reactive to *P. falciparum* infected red blood cells was immobilized onto the surface of a gold nanoparticle-modified screen-printed electrode. The assay showed good sensitivity with a linearity ranging from 10^2^ cells/mL to 10^8^ cells/mL [51]. The use of such an approach employing whole cells to detect malaria infection may help in overcoming the paucity of known biomarkers in malaria. In another study, Surface enhanced Raman spectroscopy (SERS) was applied in probing the hemozoin content of red blood cells (RBCs) and was found to be suitable for the diagnosis of malaria at low parasitemia and hemozoin concentrations in blood. It demonstrated an ultrasensitive detection limit for hemozoin at 0.00005% parasitemia level in the ring stage (2.5 parasites/µL) [60].

Ittarrat et al. developed a sensitive and specific DNA biosensor based on Quartz Crystal Microbalance (QCM) technology. The assay could significantly differentiate between *P. falciparum* and *P. vivax* in infected blood [61]. In their assay, avidin and a biotinylated probe were sequentially immobilized on a silver-based QCM. The use of silver provides a cost-effective alternative for resource-limited settings because silver-based QCM are up to ten times cheaper (USD 1.0 per tested sample) than the conventional gold-based QCM. Their sensor was stable at room temperature for up to 2 months, and could significantly differentiate malaria infected and non-infected blood by using 10.0 μL of parasite DNA at 10.0 μg/mL [61].

A class of nucleic acid molecules called aptamers have also been utilized to recognize and bind target molecules with high specificity [62]. Single-strand DNA aptamers for parasite lactate dehydrogenase (pLDH) have been identified via the Systematic Evolution of Ligands by EXponential enrichment (SELEX) that selectively binds to the target proteins with high sensitivity [63,64]. Aptasensors, based on electrochemical impedance spectroscopy (EIS), have been developed to selectively detect both *P. vivax* lactate dehydrogenase (*Pv*LDH) and *P. falciparum* lactate dehydrogenase (*Pf*LDH). In the study, a single-stranded DNA aptamer for pLDH was used as a probe to selectively detect recombinant PvLDH and PfLDH *in vitro* with detection limits of 108.5 fM and 120.1 fM, respectively. In addition, the aptasensor could detect infected blood samples from non-infected blood with detection limit of 1 parasite/µL [63]. Clearly, aptasensors serve as simple and rapid alternative methods for malaria diagnosis.

## 6. Multiplex Biomarker Detection

The use of a single disease marker is clearly of little use; thus, there is a continuing demand for diagnostic platforms that offer the potential of improved diagnostic accuracy and high-throughput capability. Multiplexed biomarker analysis enables TTAT, saves labor and offers the possibility of further miniaturization of the devices. For malaria, it is especially relevant in speciation of the infection which would guide treatment course [65], as well as minimize cost by reducing the number of required tests for accurate diagnosis.

Multiplexed detection systems have commonly used *Pf*HRP2 and pan-malarial aldolase to differentiate species in malaria infection. Nash et al. [66] have demonstrated that a gold and iron oxide magnetic nanoparticles (AuNP/mNP) system in addition to a polymer free poly (*N*-isopropylacrylamide) (pNIPAm) could enrich and detect recombinant *Pf*HRP2 and pan-malarial aldolase antigens [66]. A multiplex assay (MPA) developed by Jepsen et al. [67], using a bead-based multiplexed immunoassay system in a microplate format (Luminex xMAP® technology) to simultaneously analyze glutamate-rich protein (GLURP) antigens R0 and R2, merozoite surface protein 3 (MSP3), MSP1 hybrid and apical merozoite antigen 1 (AMA1) for screening *P. falciparum* in blood donors with suspected malaria. Their findings established a high diagnostic performance with 95.8% specificity and 90.4% sensitivity of [67]. Given the advantages associated with the MPA, notably operator-independence and reliability, the assay has the potential for routine clinical application in endemic regions.

An attempt to ease the laborious burden of screening large samples against multiple antigens by enzyme immunoassays (EIA) saw Fouda and colleagues [68] implement the suspension array technology (SAT), which employs a fluorescent labelled microsphere with diverse optical properties. Specific antigens were attached to the microsphere to produce a multiplex assay for antibody capture. They simultaneously measured antibodies against nine antigens (two variants of MSP-1, two variants of AMA-1, MSP-3, erythrocyte binding antigen 175 (EBA-175), circumsporozoite protein (CSP), ring erythrocyte surface antigen (RESA) and liver-stage antigen 1 (LSA-1)) [68]. They reported a good correlation of the SAT with enzyme-linked immunosorbent assay (ELISA), and a 10-100-fold reduction in the amounts of protein required to perform the SAT when compared with ELISA. The assay was also rapid, reproducible and required less than 1.0 μL of plasma, thus, making it ideal for finger-prick blood or in neonates where plasma volume is limited.

Ambrosino et al. [69] developed a sensitive multiplexed assay to simultaneously detect antibodies against *P. falciparum* saliva antigens with the aim of assessing the exposure to parasites or bites from the vector [69]. In total, 13 peptides derived from the parasite proteins including liver stage antigen 1 (LSA1), LSA3,, GLURP, sporozoite and liver-stage antigen (SALSA), thrombospondin-related anonymous protein (TRAP), sporozoite threonine-and asparagine-rich protein (STARP), CSP and *P. falciparum* 11.1 gene (Pf11.1) were successfully implemented. Although such assays are more popular for the evaluation of immune responses to malarial antigens, their diagnostic application in non-endemic regions where antibody titers may be much lower in exposed persons merits further research.

A high-throughput multiplex 5′ nuclease qPCR assay was developed by Reller et al. [70] to target the 18S rRNA gene (*P. falciparum*), AMA-1 gene (*P. vivax*) and the plasmepsin genes (*P. ovale*, *P. malariae* and *P. knowlesi*). The assay showed sensitivities of 95.8%, 89.5%, 94.1%, 100% and 100% for *P. falciparum*, *P. vivax*, *P. ovale*, *P. malariae* and *P. knowlesi*, respectively, versus microscopically confirmed malaria cases. The assay had detection limits for the *Plasmodium* spp. in the range of 1 to 6 parasites/μL of blood with specificities from 98.6% to 100% [70]. Further testing of the assay showed improved sensitivity by its successful identification of 11 out of 12 known malaria positive samples with undefined speciation by microscopy; hence it provided a rapid means of identifying all species of human malaria parasites [70].

## 7. Conclusions

A significant challenge to the efforts towards the global elimination of malaria is the lack of POC diagnostic methods in endemic areas for detecting parasites in asymptomatic individuals, who are the reservoirs for transmission. Despite the evolution of diagnostic methods over the past few decades, microscopy and RDTs are still the most widely used techniques even though they have low sensitivity and specificity for malaria. These challenges associated with microscopy and RDTs warrants the exploration and implementation of molecular techniques as well as biosensing-based methods for more accurate detection, quantitation and POC application. Nucleic acid-based detection methods are highly sensitive but remain confined to research laboratories because they are expensive to run and maintain; thus, rendering them unsuitable for routine applications. The justifications for a multiplexed approach, in conjunction with biosensors, for malaria diagnosis is compelling, given the high fatality rates associated with mixed infections. Multiplexed testing strategies will improve detection rates and reduce the risk of outbreaks, as well as guide treatments. Biosensing technology has an additional advantage due its suitability for off-lab situations and is ideal for POC application. It is imperative that this technology is further exploited for the development of inexpensive, ultrasensitive and field-ready assays for malaria diagnosis.

## Figures and Tables

**Table 1:** Diagnostic biomarkers for malaria.

Biomarker	Parasite species	Infection Stage	Diagnostic Method	Function/Description	Ref.
Lactate dehydrogenase (LDH)	*P. falciparum*	Trophozoite stage	Immunochromatographic assays	Metabolic enzyme in glycolytic pathway to convert pyruvate into lactate	[13,14,15]
*P. falciparum* Histidine-Rich Protein 1 (*Pf*HRP1)	*P. falciparum*	Asexual stages and gametocytes of *P. falciparum*, expressed on red blood cell membrane surface (Knob positive strains)	Immunochromatographic assays	Assist co-adherence of infected erythrocyte to venular endothelial cells	[16,17]
*P. falciparum* Histidine-Rich Protein 2 (*Pf*HRP2)	*P. falciparum*	Asexual stages and gametocytes of *P. falciparum*, expressed on red blood cell membrane surface (Knob-positive and negative strains)	Immunochromatographic assays, Enzyme-Linked Immunosorbent Assay (ELISA)	Tightly binding with glycosaminoglycans causing inhibition of antithrombin and detoxification of heme by forming hemozoin	[15,18,19,20]
*P. falciparum* Histidine-Rich Protein 3 (*Pf*HRP3)	*P. falciparum*	Asexual stages and gametocytes of *P. falciparum*, expressed on red blood cell membrane surface	Immunochromatographic assays	Function is similar to *Pf*HRP2	[21,22]
*Plasmodium* aldolase	*P. vivax* and *P. falciparum*	Asexual blood-stage	Immunochromatographic assays	Enzymatic role in the cleavage of fructose-1,6-bisphosphate into glyceraldehyde-3-phosphate and dihydroxyacetone phosphate in the glycolytic pathway	[23,24]
Hemozoin	All *Plasmodium* spp.	Intra-erythrocytic stage	Magneto-Optical Detection	Metabolite formed by polymerizing free-toxic heme after digestion of hemoglobulin by *Plasmodium*	[25]
Glutamate dehydrogenase (GDH)	*P. falciparum*	Intra-erythrocytic development	Western blotting, immunochromatographic assays	Responsible for the oxidative deamination of l-glutamate to produce α-ketoglutarate and ammonia	[26,27]

**Table 2:** Comparison of the analytical sensing parameters of molecular approaches developed for the diagnosis of malaria.

Methods	Specimen	*Plasmodium* spp.	Target	Limit of Detection	Sensitivity	Specificity	Ref.
Nested PCR	Urine, Saliva	*P. falciparum*, *P. vivax*	mitochondrial cytochrome b gene (*cytb*)	10 parasites/μL	*P.f*; S-74.2% *P.f*; U-55.1% *P.v*; S-79.2% *P.v*; U-53.3%	*P.f*; S-100% *P.f*; U-100% *P.v*; S-98.7% *P.v*; U-97.5%	[31]
Immunochromatography	Urine	*P. falciparum*	*Pf*HRP-2	-	83.75%	83.48%	[32]
Nested PCR	Blood, Saliva, Urine	*P. falciparum*	18S rRNA gene	-	B-98% S-73% U-32%	B-95% S-97% U-98%	[30]
Photo-induced electron transfer (PET)-PCR	Blood	*P. falciparum*, *P. vivax*, *P. malariae*, *P. ovale*	18S rRNA gene	*P.f*-3.2 parasites/µL *P.v*-5 parasites/µL *P.m*-3.5parasites/µL *P.o*-5.8 parasites/µL	*P.f*-**100% *P.v*-**100% *P.m*-**100% *P.o*-**100%	*P.f*-**100% *P.v*-**100% *P.m*-**100% *P.o*-**100%	[36]
Photo-induced electron transfer (PET)-PCR	Blood	*Plasmodium* spp.		3.2 parasites/μL	**92.3%	**100%	[37]
Chemiluminescent ELISA	Saliva	*P. falciparum*	*Pf*HRP-2	173 pg/mL	*100%	*100%	[38]
LAMP assay	Blood	*P. falciparum*	apicoplast genome	-	92%	97%	[39]
Non-Instrumented Nucleic Acid (NINA)-LAMP	Blood	*P. falciparum*	DNA	-	96.8%	84.3%	[40]
LAMP	Blood	*P. falciparum*	DNA	5 DNA copies/test	40–100%	100%	[41]
Microwave irradiation and LAMP	Blood	*Plasmodium* spp.	DNA	1 parasite/μL	-	-	[42]
Lab-on-chip PCR	Archival	*Plasmodium* spp.	18S rRNA gene	2 parasites/µL	97%	93.8%	[43]
Chip-based micropcr test (Truenat® Malaria)	Blood	*P. falciparum*, *P. vivax*		<5 parasites/µL	100%	100%	[44]
Isothermal recombinase polymerase amplification (RPA)	Genomic DNA	*P. falciparum*	18S rRNA gene	100 fg of genomic *P. falciparum* DNA	-	-	[45]
Realamp method.	*P.f* (3D7) culture, *P.v* (SV4), *P.m* (Uganda I), and *P.v* (Nigeria I) acquired from infected monkeys	*P. falciparum*	18S rRNA gene	1–100 p/mL	-	-	[46]
LAMP assay	Blood	*P. vivax*	α-tubulin gene	100 copies *of P. vivax* α-tubulin gene per reaction	100%	81.6%	[47]
Surface enhanced Raman spectroscopy (SERS)	Blood	*P. falciparum*	hemozoin	0.00005%&0.01% parasitemia level	-	-	[48]
Quartz Crystal Microbalance (QCM) Biosensor	Blood	*P. falciparum*, *P. vivax*	DNA	200 ng of target DNA	-	-	[49]
Biosensor (colorimetric aptasensor)	Recombinant protein biomarkers	*P. falciparum*, *P. vivax*	Recombinant *Pv*LDH & *Pf*LDH	1.25 pM (*Pv*LDH), 2.94 pM (*Pf*LDH)	-	-	[50]
Biosensor (electrochemical immunosensor)	Blood	*P. falciparum*	*P. falciparum* infected red blood cells	-	-	-	[51]

B = blood; S = saliva; U = urine; * Microscopy as gold standard (reference); ** nested PCR as gold standard (reference); *Plasmodium falciparum (P.f), Plasmodium vivax (P.v), Plasmodium malariae (P.m), Plasmodium ovale (P.o)*.
